# Cancer incidence among Swedish firefighters: an extended follow-up of the NOCCA study

**DOI:** 10.1007/s00420-019-01472-x

**Published:** 2019-08-28

**Authors:** Carolina Bigert, Jan Ivar Martinsen, Per Gustavsson, Pär Sparén

**Affiliations:** 1 Institute of Environmental Medicine, Karolinska Institutet, Solnavägen 4, 10th Floor, 113 65 Stockholm, Sweden; 2 Centre for Occupational and Environmental Medicine, Stockholm County Council, Stockholm, Sweden; 3 Department of Research, Cancer Registry of Norway, 0304 Oslo, Norway; 4 Department of Medical Epidemiology and Biostatistics, Karolinska Institutet, Nobels väg 12A, Solna, 171 77 Stockholm, Sweden

**Keywords:** Occupational exposure, Carcinogens, Smoke, Firefighters, Cancer incidence

## Abstract

**Objectives:**

To evaluate cancer incidence among Swedish firefighters and analyze risk in relation to work duration as a proxy for cumulative exposure.

**Methods:**

This cohort study is based on the Swedish component of the Nordic Occupational Cancer (NOCCA) project. The cohort includes six million people who participated in one or more of the population censuses in 1960, 1970, 1980 and 1990. The census data were linked to the Swedish Cancer Registry for the 1961–2009 period, extending a previous NOCCA follow-up time by 4 years. We identified 8136 male firefighters. SIRs were calculated using cancer incidence rates in the national population as a reference.

**Results:**

We found a statistically significant excess of non-melanoma skin cancer (SIR = 1.48, 95% CI 1.20–1.80) but no positive relationship between risk and work duration. There was a small, yet statistically significant increased risk of prostate cancer among firefighters with service times of 30 years or more. The first follow-up period (1961–1975) showed an increased risk of stomach cancer relative to the reference group, while the last period (1991–2009) showed an increased risk of non-melanoma skin cancer. There was no excess risk for all cancer sites combined (SIR = 1.03, 95% CI 0.97–1.09).

**Conclusions:**

Our results do not support an overall risk of cancer among Swedish firefighters, but a possible risk of non-melanoma skin cancer exists. The previously noted excess of prostate cancer among Swedish firefighters in NOCCA was no longer statistically significant in this extended follow-up but was present among those with the longest service times.

## Introduction

As part of their work, firefighters come in contact with smoke, dust and vehicle exhaust, all of which may expose them to a wide range of carcinogens, e.g. benzo(a)pyrene, 1,3-butadiene, benzene, cadmium, arsenic, formaldehyde, asbestos, crystalline silica dust, polychlorinated biphenyls and diesel exhaust (IARC 2010). They also work shifts which may disrupt their circadian rhythms, and as such, influence the risk of developing certain cancers.

Previous studies of cancer incidence among firefighters have reported inconsistent results regarding overall cancer risk as well as the risk of developing specific cancer types. According to a review and meta-analysis by LeMasters et al. (2006), there is convincing evidence that firefighters have an increased risk of developing specific cancer types, notably non-Hodgkin lymphoma, multiple myeloma, prostate cancer and testicular cancer. Furthermore, the results showed that firefighters may be more prone to developing other cancers such as e.g. non-melanoma skin cancer, malignant melanoma, leukemia and stomach cancer. The International Agency for Research on Cancer (IARC) concluded that the evidence was strongest for non-Hodgkin lymphoma, prostate cancer and testicular cancer, and classified “occupational exposure as a firefighter” as possibly carcinogenic to humans (group 2B) (IARC 2010).

More studies of cancer among firefighters have been published since then with varying findings. Only a few of them have applied useful exposure measures (for example, Daniels et al. 2014, 2015; Glass et al. 2016; Petersen et al. 2018a, b) and no consistent dose-response patterns have been established.

A more recent overview of the state of knowledge about firefighters and cancer was performed by Jalilian et al. (2019), including 50 papers in the review and 48 papers in the meta-analysis. They identified elevated incidence rates of several specific cancers, e.g. colon, rectal, prostate, testicular, bladder, pleural (mesothelioma) and thyroid cancer along with malignant melanoma, among firefighters, but did not show increased incidence for all cancer sites combined. The mortality rates were increased for rectal cancer and non-Hodgkin’s lymphoma (Jalilian et al. 2019).

A few previous studies have focused on cancer among Swedish firefighters. Tornling et al. (1994) performed a cohort study on 1116 firefighters who had worked in the city of Stockholm. A recent update of that study, which added 26 years of follow-up, showed an increased incidence of stomach cancer among firefighters, yet reported decreased incidence of both prostate cancer and skin-melanoma, along with an overall decreased risk of total cancer relative to the general population (Kullberg et al. 2018).

A previous study based on the Nordic Occupational Cancer (NOCCA) project included more than 16,000 firefighters from Sweden, Norway, Finland, Denmark and Iceland, and followed cancer incidence between 1961 and 2005. This study—which included the national populations as a reference—showed increased risk of adenocarcinoma of the lung, prostate cancer, non-melanoma skin cancer and skin-melanoma among firefighters, as well as a small excess risk when all cancer sites were combined. However, the study also reported that firefighters have a decreased risk of testicular cancer relative to the general population (Pukkala et al. 2014). The Swedish portion of the NOCCA reported that Swedish firefighters have an increased risk for non-melanoma skin cancer and a borderline increased risk for prostate cancer (Pukkala et al. 2014).

The aim of this study was to further evaluate cancer incidence among Swedish firefighters in NOCCA by extending the follow-up time to 2009 and assessing how work duration is related to cancer risk.

## Methods

The present study is based on the Swedish component of the NOCCA project (Pukkala et al. 2009, 2014), which includes 6 million Swedish men and women who participated in one or more of 1960, 1970, 1980 or 1990 population censuses. A previous paper that utilized the NOCCA data evaluated cancer incidence among firefighters in the Nordic countries for the 1961–2005 period (Pukkala et al. 2014). In the present study, we added 4 years to the follow-up period by extending it to 2009 and investigated how the duration of work influences cancer risk among Swedish firefighters.

The NOCCA project and information about the occupational and diagnostic coding has been described in detail elsewhere (Pukkala et al. 2009, 2014). A brief summary of the methods is presented below.

Information on occupation was available from computerized census records. The census questionnaires were self-administered and included questions about economic activity, occupation, and industry. Occupations were coded according to the Nordic Occupational Classification (NYK), a Nordic adaptation of the International Standard of Classification of Occupations (ISCO) from 1958. A person was classified as a firefighter in the census record if he/she had reported working in the occupation for more than half of the regular working hours that year.

Information on cancer incidence was obtained by linking the census records to the nation-wide Swedish Cancer Registry that started in 1958. Data regarding dates of death and emigration were obtained from the Central Population Register. In the present follow-up, the Swedish NOCCA data have been updated with cancer information until 2009. A person entered the cohort on January 1st of the year after the first available census (1960, 1970, 1980 or 1990) in which they participated, provided that they were 30–64 years old and registered as a firefighter. Person-years were then counted until the date of emigration, death or December 31st 2009. We had information on emigration until 2002.

We identified 8136 male firefighters. Female firefighters (*n* = 15) were excluded because there were too few for the analysis.

The identified firefighters were followed from 1961 (or later depending on when they entered the cohort) to 2009, and the standardized incidence ratio (SIR) was calculated with the cancer incidence rate for the national male study population used as a reference. The observed number of cancer cases and person years were stratified in 5 year age categories and 5 year calendar periods. The expected number of cancer cases was calculated by summarizing the product of person-years in each stratum and the corresponding reference rate. SIRs were calculated as the ratio of observed and expected cancer cases. For each SIR, the 95% CI was defined assuming a Poisson distribution of the observed number of cases.

Work duration was calculated as the number of years that a person had worked as a firefighter during the follow-up period. If a person had had different occupations in different censuses, we assumed that they had changed occupations in the middle of the known census years. For the 1955–1960 period, we assumed that each individual’s earlier occupation was the same as it was in 1960; similarly, occupation in 1990 was used for the 1990–2009 period. For persons attaining age 65 between two censuses or between the census in 1990 and end of follow-up, employment was assumed to end at age 65, the general retirement age in Sweden. We also conducted sensitivity analyses assuming the exposure after 1990 was only 5 or 10 years and analyses assuming exposure to stop at latest at age 55 or 60 instead of age 65. The *p* values for identified trends were calculated by modelling the SIRs using a generalized linear model (GLM) with a Poisson error structure. The unexposed group (SIR = 1) was not included in the GLM.

The cancer cases were grouped into 49 main categories and 27 diagnostic subgroups. In the present study, only the first incident cancer within a given diagnostic group was included in the analyses. For selected cancer sites, we also stratified the results by employment duration (1–9 years, 10–19 years, 20–29 years, 30 + years) to analyze the risk in relation to work duration as a proxy for cumulative exposure, and by calendar period of follow-up (1961–1975, 1976–1990, 1991–2009) for information on whether occupational exposures of relevance for the cancers appear to have varied over time. The selected cancer sites were those with more than ten cancer cases and which showed an increased overall cancer risk in the present study, a previous study of Swedish firefighers (Kullberg et al. 2018) or the earlier study of Nordic firefighters (Pukkala et al. 2014). We also included cancer sites mentioned as being of particular interest by LeMasters et al. (2006) and/or the IARC (2010), and we added mesothelioma.

## Results

Among a total of 8136 male Swedish firefighters, we observed 1483 incident cancer cases during the 1961–2009 follow-up period, which adds a further 374 cases to the previous follow-up of Swedish firefighters in NOCCA. The characteristics of the Swedish firefighter cohort and information regarding the start, end and duration of follow-up are presented in Table 1. The mean year of birth was 1940, while the mean age at the start and end of follow-up was 37 and 65, respectively. The low number of people with start of follow-up at 1971 may possibly be explained by lower recruitment of people to the firefighter occupation (as well as to other occupations within public safety and protection) in the 1960s in Sweden, combined with low birth rates in the 1930s due to the economic and political situation. The mean follow-up duration was 28 years. About 32% of the firefighters had been registered as a firefighter in only one of the four population censuses, whereas 37%, 27% and 4% were registered as a firefighter in two, three and four censuses, respectively.

**Table 1:** Characteristics of the Swedish firefighter cohort (*n* = 8136) and information on start, end and duration of the follow-up. Follow-up period 1961–2009

	*n*	Percent
Emigration and Vital status Dec 31 2009		
Emigrated	13	0.2
Dead	2314	28.4
Alive	5809	71.4
Year of birth (min: 1896, max: 1960, mean: 1940)		
< 1920	1082	13.3
1920–1929	1140	14.0
1930–1939	898	11.0
1940–1949	2424	29.8
1950–1960	2592	31.9
Start of follow-up		
1961	2290	28.2
1971	961	11.8
1981	2504	30.8
1991	2381	29.2
End of follow-up (min: 1961, max: 2009, mean: 2004)		
1961–1969	89	1.1
1970–1979	239	2.9
1980–1989	436	5.3
1990–1999	713	8.8
2000–2009	6659	81.9
Age at start of follow-up (min: 30, max: 64, mean: 37)		
30–39	6950	85.4
40–49	731	9.0
50–59	398	4.9
60–64	57	0.7
Age at end of follow-up (min: 31, max: 100, mean: 65)		
30–39	33	0.4
40–49	295	3.6
50–59	2658	32.7
60–69	2717	33.4
70+	2433	29.9
Duration of follow-up^a^ (min: 0.02, max: 49, mean: 28) (years)		
> 0–9	173	2.1
10–19	2693	33.1
20–29	2884	35.5
30–39	1394	17.1
40+	992	12.2
Number of censuses where registered as a firefighter		
1	2583	31.8
2	3029	37.2
3	2168	26.6
4	356	4.4

^a^Does not represent the number of years working as a firefighter but rather the stratified follow-up periods

The cancer incidence for specific cancer sites with at least 10 cases (as well as for testis cancer and mesothelioma since they are of specific interest) and for all cancer sites combined are presented in Table 2. We found a statistically significant excess of non-melanoma skin cancer (SIR = 1.48, 95% CI 1.20–1.80) among firefighters relative to the reference group. There was no excess risk for all cancer sites combined (SIR = 1.03, 95% CI 0.97–1.09).

**Table 2:** Cancer incidence among 8136 male Swedish firefighters. Follow-up 1961–2009

ICD-10	Cancer site	Obs	Exp	SIR	95% CI
C09–14	Pharynx	13	12.5	1.04	0.55–1.78
C15	Esophagus	13	18.4	0.71	0.38–1.21
C16	Stomach	60	55.4	1.08	0.83–1.39
C18	Colon	101	100	1.01	0.82–1.23
C19–21	Rectum, rectosigma	63	70.6	0.89	0.69–1.14
C22	Primary liver	15	16.9	0.89	0.50–1.47
C25	Pancreas	43	36.6	1.17	0.85–1.58
C32	Larynx	12	13.0	0.92	0.48–1.61
C34	Lung	110	125	0.87	0.72–1.05
	Adenocarcinoma	31	30.7	1.01	0.69–1.43
	Small cell	10	13.9	0.72	0.34–1.32
	Squamous cell	38	40.7	0.93	0.66–1.28
	Other	31	40.5	0.77	0.52–1.09
C45	Mesothelioma	7	6.30	1.11	0.45–2.29
C61	Prostate	444	420	1.06	0.96–1.16
C62	Testis	4	10.2	0.39	0.11–1.01
C64	Kidney	41	48.6	0.84	0.61–1.14
C66-68	Bladder	109	101	1.08	0.89–1.31
C43	Melanoma skin	69	56.6	1.22	0.95–1.54
C44	Non-melanoma skin	101	68.3	1.48	1.20–1.80
C70–72	Brain	38	42.5	0.89	0.63–1.23
	Glioma	18	19.2	0.94	0.56–1.48
C49	Soft tissue	15	10.3	1.46	0.82–2.41
C83, C85	Non-Hodgkin lymphoma	42	40.2	1.05	0.75–1.41
C90	Multiple myeloma	26	20.8	1.25	0.82–1.83
C91–95	Leukemia	33	34.9	0.94	0.65–1.33
	Chronic lymphatic	14	16.4	0.85	0.47–1.43
C00–99	All cancers	1483	1438	1.03	0.97–1.09

*Obs* observed number of cases, *Exp* expected number of cases, *SIR* standardized incidence ratio, *CI* confidence interval

The results from analyses stratified by duration of employment are shown in Table 3. The risk of non-melanoma skin cancer was significantly elevated among firefighters who had worked 10–19 years (SIR = 1.82, 95% CI 1.21–2.62) and 20–29 years (SIR = 1.56, 95% CI 1.09–2.17). However, the risk was non-significantly elevated among firefighters who worked 30 years or more. There was a small, yet statistically significant, excess of prostate cancer among firefighters who had worked 30 years or more (SIR = 1.14, 95% CI 1.01–1.29), while all of the other three work duration categories showed SIR values that were below one. Trend analyses found no positive, statistically significant association between risk and employment duration for any of the studied cancer sites or all of the cancer sites combined. Sensitivity analyses assuming the exposure after 1990 was only 5 or 10 years showed similar risk estimates as in Table 3, but the risk of multiple myeloma was now statistically significantly increased among firefighters who worked 30 years or more (SIR = 1.89, 95% CI 1.03–3.17, both when assuming the exposure to stop 1995 and in 2000). Additional analyses assuming exposure to stop at latest at age 55 or 60 instead of age 65 also showed similar results as in Table 3, although the risk of prostate cancer among those who had worked as a firefighter for 30 years or more was no longer statistically significantly elevated.

**Table 3:** Observed number of cases for selected cancer sites and standardized incidence ratio among 8136 male Swedish firefighters, by duration of employment. Follow-up 1961–2009

ICD-10	Cancer site	1–9 years			10–19 years			20–29 years			30 + years			*p* trend
		Obs	SIR	95% CI	Obs	SIR	95% CI	Obs	SIR	95% CI	Obs	SIR	95% CI	
C16	Stomach	4	1.43	0.39–3.66	22	1.23	0.77–1.86	18	1.00	0.59–1.57	16	0.97	0.55–1.58	*0.75*
C34	Lung	3	1.03	0.21–3.01	33	1.06	0.73–1.48	34	0.85	0.59–1.18	40	0.78	0.56–1.06	*0.10*
	Adenocarcinoma	1	2.59	0.07–14.4	8	1.32	0.57–2.60	6	0.65	0.24–1.41	16	1.06	0.61–1.72	*0.94*
C45	Mesothelioma	1	13.68	0.35–76.2	0	0.00	0.00–2.80	3	1.46	0.30–4.28	3	1.04	0.21–3.04	*0.85*
C61	Prostate	2	0.50	0.06–1.81	76	0.94	0.74–1.18	114	0.98	0.81–1.17	252	1.14	1.01–1.29	*0.13*
C43	Melanoma skin	0	0.00	0.00–2.30	17	1.24	0.72–1.98	27	1.42	0.94–2.07	25	1.11	0.72–1.65	*0.11*
C44	Non-melanoma skin	0	0.00	0.00–3.70	28	1.82	1.21–2.62	35	1.56	1.09–2.17	38	1.28	0.91–1.76	< 0.01*
C83, C85	Non-Hodgkin lymphoma	1	0.88	0.02–4.89	12	1.10	0.57–1.93	17	1.17	0.68–1.87	12	0.88	0.45–1.53	*0.90*
C90	Multiple myeloma	0	0.00	0.00–7.24	4	0.77	0.21–1.96	8	1.17	0.51–2.31	14	1.70	0.93–2.85	*0.11*
C00–99	All cancers	27	0.81	0.53–1.18	349	1.01	0.92–1.14	461	1.03	0.94–1.13	646	1.04	0.97–1.13	*0.19*

Italic values indicate *p* trend; < 0.05 was considered statistically significant

*Obs* observed number of cases, *SIR* standardized incidence ratio, *CI* confidence interval

*There was a statistically significant trend of decreasing risk with increasing employment duration

The results from analyses stratified by follow-up period are presented in Table 4. We found a statistically significantly increased risk of stomach cancer during the first follow-up period (1961–1975; SIR = 1.85, 95% CI 1.06–3.00), but not in the 1976–1990 and 1991–2009 periods. There was an increased risk of non-melanoma skin cancer during the last follow-up period (1991–2009; SIR = 1.55, 95% CI 1.23–1.92).

**Table 4:** Observed number of cases for selected cancer sites and standardized incidence ratios among 8136 male Swedish firefighters, by time period. Follow-up 1961–2009

ICD-10	Cancer site	1961–1975			1976–1990			1991–2009		
		Obs	SIR	95% CI	Obs	SIR	95% CI	Obs	SIR	95% CI
C16	Stomach	16	1.85	1.06–3.00	22	1.16	0.73–1.76	22	0.79	0.49–1.19
C34	Lung	11	0.94	0.47–1.68	32	0.84	0.58–1.19	67	0.88	0.68–1.12
	Adenocarcinoma	2	1.50	0.18–5.40	6	0.87	0.32–1.90	23	1.02	0.65–1.53
C45	Mesothelioma	0	0.00	0.00–19.0	2	1.29	0.16–4.67	5	1.10	0.36–2.56
C61	Prostate	8	0.68	0.29–1.34	77	1.09	0.86–1.36	359	1.06	0.95–1.18
C43	Melanoma skin	5	1.56	0.51–3.65	14	1.10	0.60–1.85	50	1.23	0.91–1.62
C44	Non-melanoma skin	2	0.87	0.11–3.16	15	1.28	0.71–2.11	84	1.55	1.23–1.92
C83, C85	Non-Hodgkin lymphoma	1	0.35	0.01–1.97	10	0.84	0.40–1.54	31	1.22	0.83–1.73
C90	Multiple myeloma	2	1.17	0.14–4.21	6	1.07	0.39–2.32	18	1.34	0.79–2.11
C00–99	All cancers	95	0.96	0.78–1.18	352	1.03	0.93–1.15	1036	1.04	0.97–1.10

*Obs* observed number of cases, *SIR* standardized incidence ratio, *CI* confidence interval

## Discussion

This is an extended follow-up of cancer incidence in a cohort of firefighters in the Swedish part of the NOCCA-study. We found that total cancer incidence during 1961–2009 was no higher among Swedish firefighters than what was observed for the national population. However, we did find that Swedish firefighters have an increased risk of non-melanoma skin cancer. Although the risk did not increase with work duration (on the contrary, there was a statistically significant trend of decreasing risk with increasing work duration), a statistically significant excess of non-melanoma skin cancer was found in two out of the four work duration categories. We also found a statistically significantly increased risk of prostate cancer among those who had worked as a firefighter for 30 years or more and a statistically significantly elevated risk of stomach cancer during the first follow-up period (1961–1975). There was also a tendency for increased risk of multiple myeloma among firefighters with a work duration of 30 years or more.

When our results are compared with the previous NOCCA study—which included firefighters from all of the Nordic countries—some similar patterns can be discerned. However, the overall significant increases in prostate cancer, skin melanoma and lung adenocarcinoma incidence that were found in the Nordic study (Pukkala et al. 2014) were not identified in our follow-up of Swedish firefighters. This is potentially due to a smaller number of cases and thus, a smaller statistical power for detecting small excesses in cancer risk when the study was limited to Swedish firefighters. However, it could also be that Swedish firefighters do not have an increased risk for these cancers. The risk estimates for lung adenocarcinoma and prostate cancer provided by our analyses—which were based on 31 and 444 observed cancer cases, respectively—were very close to one. The risk estimate for melanoma skin cancer, which was 1.22 with a 95% CI of 0.95–1.54 and based on 69 observed cases, indicates a possible increased risk among Swedish firefighters, yet not one that statistically differs from that of the general population.

We did not find an increased risk for non-Hodgkin’s lymphoma, mesothelioma or testis cancer, that has previously been associated with firefighting (Jalilian et al. 2019). These findings do not rule out a possible risk for these cancers among Swedish firefighters, since the numbers are too small to detect small excesses, especially for mesothelioma and testis cancer where analyses were based on seven and four observed cases, respectively.

A previous update of a cohort of firefighters in Stockholm, Sweden, revealed decreased risks of skin-melanoma and prostate cancer, along with a risk estimate of less than one for lung cancer (Kullberg et al. 2018). Thus, our result that Swedish firefighters do not have an increased risk of lung cancer relative to the general population is in line with the results of Kullberg et al. and a previous study in which firefighters from Europe, Canada, New Zealand, and China did not show excess of lung cancer overall or of adenocarcinoma of the lung after adjustment for smoking habits (Bigert et al. 2016). The previous cohort study of Stockholm firefighters showed a clearly increased risk of stomach cancer that was independent of both work duration and the starting year of employment (Kullberg et al. 2018). Thus, our finding that firefighters do not have an overall increased risk of stomach cancer is somewhat surprising, although we did find an increased risk during the first follow-up period (1961–1975; SIR = 1.85, 95% CI 1.06–3.00); this result indicates that occupational exposure may have been more relevant to stomach cancer at earlier time periods, such as the 1940s and 1950s. The difference in the mean year of birth (1925 in the Stockholm firefighter cohort and 1940 in the Swedish NOCCA firefighter cohort) of firefighters included in these two studies supports this theory. On the other hand, a Danish study found increased mortality from stomach cancer among full-time firefighters with a mean year of birth of 1956, indicating that work in more recent time periods also has the potential to increase the risk (Petersen et al. 2018b). Possible occupational exposures related to the risk of stomach cancer in firefighters are asbestosis (Cogliano et al. 2011) and crystalline silica (Lee et al. 2016), both of which are associated with work involving dust.

Soot and solar radiation, both of which are classified as carcinogenic to humans by the IARC, may explain our finding of increased risk of non-melanoma skin cancer among firefighters. Soot is composed of a mix of different chemicals such as metals, PAHs and quartz (IARC 1985, 1987). Firefighters may be occupationally exposed to soot via both inhalation and skin contact, especially if protective equipment is not fully used. We do not know the extent to which firefighters were exposed to solar radiation during work or during their spare time, but the risk of developing non-melanoma skin cancer increases with exposure to solar radiation. It is also possible that combined exposure to soot and solar radiation could have increased the risk of non-melanoma skin cancer. Since firefighters need to pass regular medical check-ups throughout their employment it cannot be ruled out that skin cancer has been more easily detected among firefighters than in the general population, although the check-ups do not specifically include cancer screening.

Our finding that firefighters with long work duration have an increased risk of prostate cancer is in line with findings from several studies which found that firefighters have an increased risk of prostate cancer or that prostate cancer risk is associated with longer service (Glass et al. 2016; Harris et al. 2018; LeMasters et al. 2006; Petersen et al. 2018a; Jalilian et al. 2019). A review and meta-analysis on prostate cancer among firefighters showed small, but statistically significant excesses (Sritharan et al. 2017). Even though a recent study of Danish firefighters found slightly elevated overall prostate cancer incidence among firefighters when compared to other employees, the analyses neither revealed an exposure–response relationship for prostate cancer nor found a significantly elevated risk of prostate cancer for any of the employment duration categories (Petersen et al. 2018a). A study of Swedish firefighters in Stockholm yielded conflicting results, as the results showed decreased overall incidence of prostate cancer among firefighters as well as among those that had served for more than 30 years (Kullberg et al. 2018). Shift work is one of the few occupational exposures that has been linked to prostate cancer risk (IARC 2010); shift and night work is common among firefighters, although we do not have information on the shift schedules or sleeping opportunities during night shifts for the firefighters in this study.

A strength of this study is that it is relatively large, comprising all Swedish men who participated in the national population censuses, as well as including more than 8000 firefighters. Computerized records—based on census questionnaires—provided information on occupation. Another advantage is that the Swedish Cancer Registry—which has a coverage of about 96%—enabled us to reliably study cancer incidence. Furthermore, the study included a long follow-up time with a mean of 28 years. However, the study is too small to provide enough statistical power for the detection of small excesses in cancer risk of rare cancers. Also, we did not have information about potential confounders such as smoking or other individual lifestyle factors. Another limitation is that there was a 10 year gap between censuses, which, when considering occupational information, means that there is a real risk that people have changed their occupation in between. This could introduce non-differential exposure misclassification and bias the association between exposure and outcome. There is also a risk that we overestimated the active firefighting time in analyses of work duration since the firefighters may have quitted earlier than 2009 (or age 65) and they may also be likely to shift from active firefighting duty as they age. However, sensitivity analyses generally revealed small differences in results when using more strict cut-offs for work duration. A previous study on Swedish firefighters showed an average time in the profession of 26 years (median 30 years) but provided no information on whether the work tasks changed during working life (Kullberg et al. 2018). Since the firefighters were compared to a reference group of Swedish men, there is also a risk of the healthy worker effect, even though this effect would probably be smaller for cancers than for other health conditions, e.g. cardiovascular disease.

Regarding the generalizability of the results, there may be major differences between countries in occupational health and safety regulations, work activities and schedules for firefighters, the extent to which protective equipment is used and if medical checks are carried out. A firefighter’s exposure may have also differed between time periods and depended on whether they work in a large city or a rural area (Fritschi and Glass 2014). In Sweden, firefighters and employers are aware of the risk of the profession and it is currently mandatory that firefighters attend regular medical checks to assess their eligibility.

## Conclusion

In this cohort of Swedish firefighters, we found an overall increased incidence of non-melanoma skin cancer among firefighters that was also evident during the last follow-up period (1991–2009), but no increased incidence for all of the cancer sites combined. We also found increased incidence of prostate cancer among those who had worked as a firefighter for 30 years or more, along with increased incidence of stomach cancer during the first follow-up period (1961–1975). Exposure to soot and solar radiation are potential explanations for the increased risk of non-melanoma skin cancer, and shift work is a possible explanation for the increased prostate cancer risk. However, we do not have individual information on these exposures or confounders for the firefighters included in this study. The association between occupational exposure and the increased risks of non-melanoma skin cancer and prostate cancer is therefore uncertain. Our results provide some support to previous findings of increased risk of prostate cancer among firefighters with long service, stomach cancer incidence among firefighters who started in the profession further back in time, and non-melanoma skin cancer incidence among firefighters with a more recent employment history.
